# Association Between Intraoperative Oliguria and Postoperative Acute Kidney Injury in Patients Undergoing Hepatobiliary and Pancreatic Surgery Within an Enhanced Recovery After Surgery Protocol: A Retrospective Cohort Study

**DOI:** 10.3390/jcm15135240

**Published:** 2026-07-04

**Authors:** Hwa-Young Jang, Hyun-Jung Kwon, Yong-Hee Park, Sung-Moon Jeong, Yeon Ju Kim, Hye-Mee Kwon

**Affiliations:** Department of Anesthesiology and Pain Medicine, Asan Medical Center, University of Ulsan College of Medicine, 88 Olympic-ro 43-gil, Songpa-gu, Seoul 05505, Republic of Korea; janghy315@amc.seoul.kr (H.-Y.J.); kwonhj@amc.seoul.kr (H.-J.K.); d231427@amc.seoul.kr (Y.-H.P.); anesjsm@amc.seoul.kr (S.-M.J.)

**Keywords:** acute kidney injury, intraoperative, oliguria, enhanced recovery after surgery, anesthesia, perioperative care

## Abstract

**Background/Objectives**: Intraoperative oliguria has long been considered a marker of impaired renal perfusion, but its prognostic value for postoperative acute kidney injury (AKI) remains controversial, particularly within Enhanced Recovery After Surgery (ERAS) protocols. We investigated the association between intraoperative oliguria and postoperative AKI in patients undergoing major hepatobiliary and pancreatic surgery within an ERAS protocol. **Methods**: Patients who underwent major hepatobiliary and pancreatic surgery within an institutional ERAS protocol were retrospectively analyzed. Intraoperative oliguria was defined as urine output < 0.3 mL kg^−1^ h^−1^. Postoperative AKI was defined according to the KDIGO serum creatinine criterion within 7 days after surgery. The association was assessed using multivariable logistic regression and sensitivity analyses were performed using an alternative oliguria threshold of <0.5 mL kg^−1^ h^−1^ and incorporating additional surgical covariates. **Results**: Among 816 patients, intraoperative oliguria occurred in 51 (6.3%), and postoperative AKI developed in 60 (7.4%). AKI incidence did not differ between the oliguria and non-oliguria groups (11.8% vs. 7.1%, *p* = 0.332), and median intraoperative urine output was comparable between the AKI and non-AKI groups (0.7 [0.5–1.1] vs. 0.8 [0.6–1.4] mL kg^−1^ h^−1^, *p* = 0.069). In multivariable analysis, intraoperative oliguria was not independently associated with AKI (OR 1.68, 95% CI 0.60–4.01; *p* = 0.276). Oral carbohydrate loading, thoracic epidural analgesia, and total intraoperative fluid volume were not associated with AKI. Results were consistent across both sensitivity analyses. **Conclusions**: In patients undergoing hepatobiliary and pancreatic surgery within the ERAS protocol, intraoperative oliguria was not associated with postoperative AKI, although modest association cannot be excluded given the limited number of AKI and oliguria events. These findings suggest that intraoperative urine output alone may not be a reliable indication for additional fluid administration, and larger prospective studies are needed to confirm this.

## 1. Introduction

Intraoperative oliguria has traditionally been regarded as a marker of renal hypoperfusion and a potential precursor of acute kidney injury (AKI). However, the prognostic significance of intraoperative oliguria for postoperative AKI remains a matter of debate. While some studies have reported a significant association between intraoperative oliguria and postoperative AKI [1,2], others have failed to demonstrate such a relationship [3,4,5], suggesting that low urine output during surgery may not consistently reflect renal dysfunction.

This issue is particularly relevant in patients managed under the Enhanced Recovery After Surgery (ERAS) protocol [6,7,8,9], in which intraoperative oliguria is not uncommon [10]. Several components of the ERAS protocol can contribute to reduced urine output, including zero-balance fluid management with an increased risk of relative hypovolemia, sympathetic blockade and hypotension associated with thoracic epidural analgesia, vasoconstriction from the relatively liberal use of vasopressors, and the renal effects of nonsteroidal anti-inflammatory drugs as part of multimodal analgesia [11,12,13]. At the same time, other elements of the protocol—such as preoperative carbohydrate loading and goal-directed fluid optimization—are intended to preserve euvolemia and renal perfusion, potentially offsetting the risk of AKI [14,15]. Whether these counterbalancing effects translate into a clinically meaningful change in the relationship between intraoperative oliguria and postoperative AKI remains unclear.

We therefore conducted this study to investigate the association between intraoperative oliguria and postoperative AKI in patients undergoing major hepatobiliary and pancreatic surgery within an ERAS protocol. The primary outcome was the incidence of postoperative AKI, defined according to the Kidney Disease: Improving Global Outcomes (KDIGO) criteria.

## 2. Materials and Methods

### 2.1. Study Design, Setting, and Population

This study was approved by the Institutional Review Board of the study center (Approval no. 2024-1036) on 29 August 2024, with a waiver of written informed consent. The study was conducted in accordance with the Strengthening the Reporting of Observational Studies in Epidemiology (STROBE) guideline.

Patients who underwent major hepatobiliary and pancreatic surgery within the ERAS protocol between July 2018 and December 2023 were included. Eligible procedures included pancreaticoduodenectomy, total pancreatectomy, distal pancreatectomy, bile duct resection, and hepatic resection, all performed via laparotomy. For patients who underwent multiple surgeries during the study period, only the index (first) procedure was included. Exclusion criteria were age < 18 years, incomplete surgery due to distant metastasis, pre-existing chronic kidney disease defined as a glomerular filtration rate below 60 mL min^−1^ 1.73 m^−2^ for more than 3 months prior to surgery, intraoperative diuretic administration, and missing medical records.

### 2.2. Anesthetic and Analgesic Management Based on the ERAS Protocol

Per the institutional ERAS protocol, preoperative oral carbohydrate loading was administered as follows: 200 mL of Encover solution (JW Pharmaceutical, Seoul, Republic of Korea; 31.24 g carbohydrates, 1 kcal mL^−1^) the night before surgery, and 200 mL of Nucare NONPO (Daesang, Seoul, Republic of Korea; 12.8% carbohydrate, 1 kcal mL^−1^) two hours before anesthesia induction [16]. Total intravenous anesthesia was maintained with target-controlled infusion of propofol and remifentanil, titrated to a bispectral index between 40 and 60.

The FloTrac system (Edwards Lifesciences, Irvine, CA, USA) was used to monitor hemodynamic parameters and volume status. Zero-balance fluid management was applied, with a balanced crystalloid solution administered at 1–3 mL kg^−1^ h^−1^, and additional 100 mL boluses given for blood loss [17,18]. Intraoperative RBC transfusion was performed at the discretion of the attending anesthesiologist, generally at a hemoglobin threshold of <7 g dL^−1^ in hemodynamically stable patients and <8 g dL^−1^ in patients with significant cardiovascular comorbidity [19]. The target mean blood pressure (MBP) was ≥65 mmHg, and vasopressors were administered at the discretion of the anesthesiologist.

A thoracic epidural catheter was placed under ultrasound guidance the day before surgery. Twenty minutes before skin incision, a 5 mL bolus of 0.15% ropivacaine was administered through the catheter to attenuate surgical stimuli. Continuous infusion of 0.12% ropivacaine combined with sufentanil (0.6–0.8 μg mL^−1^) was initiated at 3–4 mL h^−1^ within one hour of the initial bolus and continued for two postoperative days, with patient-controlled boluses of 2 mL allowed every 20 min. As part of multimodal analgesia, 1 g of intravenous acetaminophen was given before incision [20], and 50 mg of intravenous dexketoprofen was administered twice daily postoperatively. Additional components of the ERAS protocol [8,9] are detailed in Supplementary Material (Table S1).

### 2.3. Data Collection

Patient data were retrospectively retrieved from the institutional electronic medical record system, including baseline characteristics, laboratory values, intraoperative variables, and postoperative outcomes. Baseline characteristics included age, sex, body mass index (BMI), comorbidities (diabetes, hypertension, ischemic cardiac disease and cerebrovascular disease), preoperative medications (renin–angiotensin system-acting agents and diuretics), and American Society of Anesthesiologists (ASA) physical status. Preoperative laboratory data included hemoglobin, serum creatinine (sCr), and albumin. Surgical variables included duration and type of surgery categorized as pancreatic resection, liver and bile duct surgery, or pancreaticoduodenectomy involving resection of the duodenum and proximal small bowel as an integral component of the procedure, as well as use of oral carbohydrate loading and thoracic epidural analgesia. Intraoperative hemodynamic data included the lowest intraoperative MBP and the stroke volume variation recorded at the end of surgery using the FloTrac system.

Variables related to intraoperative volume status were also collected, including total volumes of crystalloid and colloid, RBC transfusion, urine output, estimated blood loss (EBL), fluid balance, and vasopressor use. EBL was estimated using a hemoglobin-based formula, calculated as the product of estimated blood volume and the difference between initial and final intraoperative hemoglobin concentrations, divided by the mean hemoglobin concentration; negative values, which may arise from hemodilution due to intraoperative fluid administration, were set to zero. Fluid balance was defined as the difference between total intraoperative fluid input and output. Intraoperative urine output was calculated by dividing the total urine output by the duration of surgery and the patient’s body weight. Intraoperative oliguria was defined as an average urine output of less than 0.3 mL kg^−1^ h^−1^ [1,5]. Twenty-four patients were excluded due to missing medical records, primarily involving incomplete intraoperative monitoring data. No missing values were present in the variables used for the final analysis.

### 2.4. Outcome

The primary outcome was the incidence of postoperative AKI, defined according to the KDIGO criteria as an increase in sCr of ≥0.3 mg dL^−1^ within 48 h or ≥1.5 times baseline within 7 days after surgery [21]. Baseline sCr was the most recent preoperative value.

AKI stages were defined as follows: stage 1, an increase in sCr of 0.3 mg dL^−1^ or to 1.5 times baseline; stage 2, an increase to 2.0–2.9 times baseline; and stage 3, an increase to ≥3.0 times baseline, an absolute sCr value of ≥4.0 mg dL^−1^, or initiation of renal replacement therapy. AKI occurring within 48 h after surgery was classified as early-onset, and AKI occurring between 48 h and 7 days postoperatively as late-onset [22].

### 2.5. Statistical Analyses

All analyses were performed using R software, version 4.3.2 (R Foundation for Statistical Computing, Vienna, Austria). Continuous variables are presented as mean (SD) or median (IQR) and were compared using Student’s *t*-test or the Mann–Whitney U test, depending on data distribution. Categorical variables are presented as frequencies (percentages) and were compared using the χ^2^ test or Fisher’s exact test. A *p*-value of <0.05 was considered statistically significant.

To assess the association between intraoperative oliguria (urine output < 0.3 mL kg^−1^ h^−1^) and postoperative AKI, univariable and multivariable logistic regression analyses were performed. Candidate variables included age, sex, BMI, diabetes, hypertension, ASA physical status, oral carbohydrate loading, thoracic epidural analgesia, oliguria, preoperative sCr, intraoperative vasopressor use, total intraoperative fluid volume, and RBC transfusion. Variables with *p* < 0.1 in the univariable analysis were entered into the multivariable model using a backward elimination approach. Oliguria was retained in the multivariable model regardless of its univariable *p*-value, as it was the primary exposure of interest. The final multivariable model included 6 variables (events-per-variable ratio = 10). Collinearity among covariates was assessed using variance inflation factors (VIFs); for categorical variables with more than two levels, the generalized VIF (GVIF^(1/(2 × Df))) was reported. Results are presented as odds ratios (ORs) with 95% confidence intervals (CIs).

To evaluate the robustness of the primary findings, two sensitivity analyses were conducted. The first repeated the multivariable model using a clinically established alternative oliguria threshold of <0.5 mL kg^−1^ h^−1^. The second incorporated additional surgical covariates, including surgery type, operative duration, lowest intraoperative MBP, and RBC transfusion, to account for potential confounding by surgical complexity.

The relationship between intraoperative urine output and the probability of AKI was further examined using unadjusted restricted cubic spline regression with three knots, which allowed modelling of potential non-linear associations, for descriptive purposes. The spline curve was plotted with 95% CIs to illustrate trends and variability across the range of observed urine output values.

## 3. Results

### 3.1. Patient Characteristics

**Figure 1:**
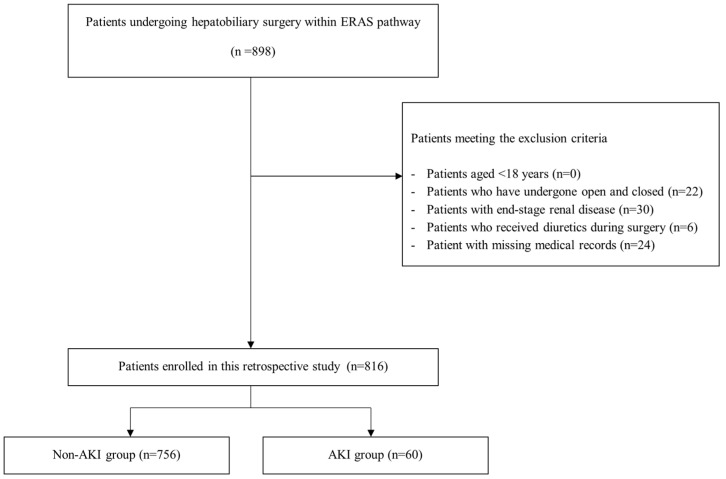
Study flow chart.

Of the 898 patients who underwent hepatobiliary and pancreatic surgery within the ERAS protocol during the study period, 82 were excluded (Figure 1), leaving 816 patients for analysis. Baseline characteristics of patients with and without intraoperative oliguria are shown in Table 1. Intraoperative oliguria occurred in 51 patients (6.3%). Compared with the non-oliguria group, patients in the oliguria group had a higher BMI (25.0 [22.6–26.7] vs. 23.3 kg m^−2^ [21.2–25.5], *p* = 0.018), and higher preoperative sCr (0.83 [0.68–0.99] vs. 0.77 [0.64–0.90] mg dL^−1^, *p* = 0.019).

### 3.2. Comparison Between the Oliguria and Non-Oliguria Groups

Intraoperative variables and AKI incidence in the two groups are summarized in Table 2. The oliguria group received a smaller volume of crystalloid (6 [5–8] vs. 7 [5–9] mL kg^−1^ h^−1^, *p* = 0.002), and had lower estimated blood loss (0 [0–329] vs. 124 [0–502] mL, *p* < 0.001). The incidence of AKI did not differ between the oliguria and non-oliguria groups (11.8% vs. 7.1%, *p* = 0.332), and neither did the distribution of AKI severity stages or the timing of AKI onset.

**Figure 2:**
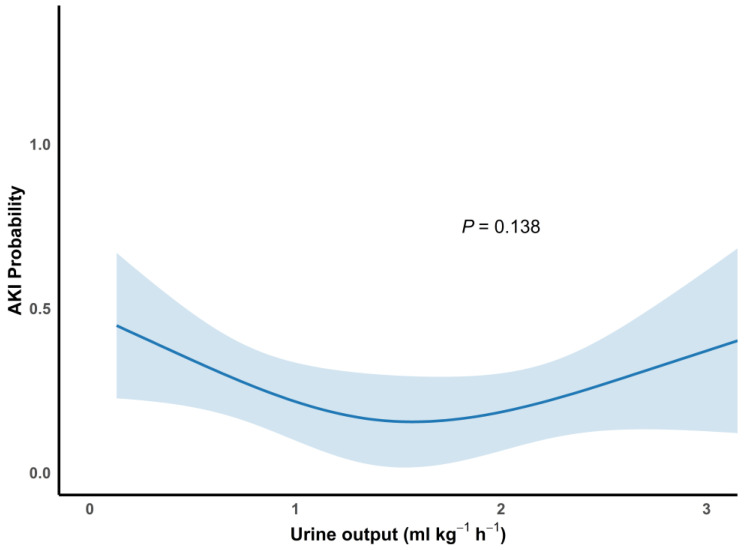
Cubic spline curves. Cubic spline curves show the unadjusted relationship between intraoperative urine output (range: 0 to 3 mL kg^−1^ h^−1^) and the probability of AKI. Shaded areas represent 95% CI. AKI, acute kidney injury; 95% CI, 95% confidence interval.

The unadjusted relationship between intraoperative urine output and AKI probability is shown in Figure 2. The cubic spline curve was relatively flat across the lower range of urine output, with no clear change in AKI probability, and the overall association was not statistically significant (*p* = 0.138).

### 3.3. Comparison Between the AKI and Non-AKI Groups

Baseline and intraoperative variables according to AKI status are shown in Table 3. Postoperative AKI developed in 60 patients (7.4%): 48 (5.9%) were stage 1, 7 (0.9%) stage 2, and 5 (0.6%) stage 3. Patients who developed AKI were more often male (81.7% vs. 60.1%, *p* = 0.001), and more frequently had hypertension (60.0% vs. 43.5%, *p* = 0.019). They also had a longer operative time (280.5 [232.0–358.0] vs. 251.5 [201.0–304.0] min, *p* = 0.001), a higher proportion of liver and bile duct surgery or pancreaticoduodenectomy (*p* = 0.003), more frequent RBC transfusion (23.3% vs. 10.6%, *p* = 0.006), lower intraoperative MBP (57 [53–62] vs. 60 [56–64] mmHg, *p* = 0.005), and greater stroke volume variation (12 [9–16] vs. 10 [8–13] %, *p* = 0.013).

Intraoperative urine output did not differ significantly between the AKI and non-AKI groups (0.7 [0.5–1.1] vs. 0.8 [0.6–1.4] mL kg^−1^ h^−1^, *p* = 0.069). The proportion of patients meeting an oliguria threshold of <0.3 mL kg^−1^ h^−1^ (10.0% vs. 6.0%, *p* = 0.332) or <0.5 mL kg^−1^ h^−1^ (28.3% vs. 19.4%, *p* = 0.137) was also comparable between the two groups. Other intraoperative fluid management variables did not differ between the groups, except that the AKI group received RBC transfusion (23.3% vs. 10.6%, *p* = 0.006) and vasopressors (78.3% vs. 63.4%, *p* = 0.028) more frequently.

### 3.4. Risk Factors for Postoperative AKI

In multivariable logistic regression analysis (Table 4), intraoperative oliguria was not associated with postoperative AKI (OR 1.68, 95% CI 0.60 to 4.01; *p* = 0.276). Independent risk factors were female sex (OR 0.28, 95% CI 0.13 to 0.54; *p* < 0.001), BMI (OR 1.09, 95% CI 1.01 to 1.18; *p* = 0.031), and intraoperative RBC transfusion (OR 2.99, 95% CI 1.49 to 5.79; *p* = 0.001). VIF values for all variables in the model ranged from 1.04 to 1.13, indicating no collinearity. Preoperative oral carbohydrate loading, thoracic epidural analgesia, and total intraoperative fluid volume were not associated with AKI in the univariable analysis.

The primary finding was consistent across both sensitivity analyses. When oliguria was redefined using a threshold of <0.5 mL kg^−1^ h^−1^, it remained non-significantly associated with postoperative AKI (OR 1.45, 95% CI 0.76 to 2.66; *p* = 0.239). In the second sensitivity analysis incorporating surgery type, operative duration, lowest MBP, and RBC transfusion as additional covariates, oliguria was also not independently associated with AKI (OR 1.12, 95% CI 0.37 to 2.81; *p* = 0.827). GVIF^(1/(2 × Df)) values in this model ranged from 1.01 to 1.09, confirming the absence of collinearity.

## 4. Discussion

In this retrospective study of 816 patients undergoing major hepatobiliary and pancreatic surgery within an ERAS protocol, intraoperative oliguria was not statistically associated with postoperative AKI. The overall incidence of AKI was 7.4%, and neither the incidence nor the severity of AKI differed between patients with and without intraoperative oliguria. Intraoperative urine output was also comparable between the AKI and non-AKI groups. In multivariable analysis, intraoperative oliguria was not independently associated with postoperative AKI; female sex was associated with a lower risk, while higher BMI and intraoperative RBC transfusion were associated with an increased risk. Oral carbohydrate loading, thoracic epidural analgesia, and the total volume of intraoperative fluid were also not associated with AKI risk. These findings should be interpreted with caution given the relatively small number of events, and further studies with larger sample sizes are warranted to draw more definitive conclusions.

The prognostic value of intraoperative oliguria for postoperative AKI has long been a matter of debate. Although urine output has traditionally been regarded as a marker of renal perfusion, it is influenced by a number of non-renal factors, including the surgical stress response, antidiuretic hormone release, anesthesia depth, and intraoperative fluid handling [4,23]. Studies on perioperative volume kinetics further suggest that oliguria during general anesthesia often reflects slow fluid distribution and reduced clearance of administered fluids rather than true renal hypoperfusion [24,25]. Consistent with this concept, several cohort studies have failed to demonstrate an independent association between intraoperative oliguria and postoperative AKI even at a threshold of <0.3 mL kg^−1^ h^−1^ [3,5], whereas others have reported a significant association at the same threshold [1,2]. Our findings extend this body of evidence by showing that, in patients managed within an ERAS protocol for hepatobiliary or pancreatic surgery, intraoperative oliguria was not statistically associated with postoperative AKI.

The ERAS protocol comprises components that may act in opposing directions with respect to intraoperative oliguria and AKI [12,13,15]. In our cohort, oral carbohydrate loading, thoracic epidural analgesia, and total intraoperative fluid volume were not associated with AKI, which would be compatible with the possibility that the counterbalancing components of the protocol contribute to overall hemodynamic stability. A previous study in colorectal surgery patients managed within an ERAS protocol reported a higher incidence of AKI in the oliguria group [10], in contrast to our findings. This discrepancy may reflect differences in surgical context: hepatobiliary and pancreatic procedures involve greater operative complexity and substantially larger blood loss, and a relatively hypovolemic state is often deliberately maintained to minimize bleeding [26]. The proportion of patients receiving intraoperative RBC transfusion was also markedly higher in our cohort than in the colorectal ERAS literature [10]. In addition, laparoscopic techniques are widely used in colorectal surgery but less so in hepatobiliary and pancreatic surgery, and pneumoperitoneum has been reported to impair renal blood flow during laparoscopic procedures [27]. These differences suggest that the relationship between intraoperative oliguria and postoperative AKI within an ERAS protocol may not be uniform across surgical specialties. In our setting, the lack of a statistically significant association between intraoperative oliguria and postoperative AKI raises the question of whether reflexive fluid administration in response to low urine output is warranted. Such a practice could conflict with the zero-balance fluid principle of the ERAS protocol and may expose patients to the risks of fluid overload, including impaired gastrointestinal recovery and prolonged hospital stay [28], without a clear renal benefit.

Several factors other than oliguria were independently associated with postoperative AKI. Female sex was associated with a lower risk, in line with previous reports suggesting a renoprotective effect of sex hormones, possibly mediated through modulation of the cellular response to ischemia–reperfusion injury [29,30]. Higher BMI was also associated with an increased risk, consistent with prior observations that both underweight and overweight patients carry a higher risk of postoperative AKI than those with normal BMI [31]. The strongest association was with intraoperative RBC transfusion. Possible mechanisms include hemolysis of transfused erythrocytes with release of free hemoglobin and iron, oxidative stress, and microcirculatory dysfunction [32,33]. It is also plausible that transfusion serves as a marker of greater intraoperative blood loss and hemodynamic instability rather than acting as a direct renal insult, and the present data cannot distinguish between these possibilities.

This study has several limitations. First, as a single-center retrospective study, our findings may be influenced by unmeasured confounding and may have limited generalizability to institutions with different patient populations or ERAS practices, although we attempted to mitigate the former through multivariable adjustment. Second, with only 60 AKI events and 51 patients with intraoperative oliguria, the study was likely underpowered to detect a small but clinically relevant association, and the null finding should not be interpreted as evidence of absence of effect. Third, AKI was defined using the KDIGO serum creatinine criterion only [21]; the urine output criterion was deliberately excluded to avoid circular reasoning, given that intraoperative oliguria itself was the primary exposure of interest. However, this approach may underestimate the true incidence of postoperative AKI, as some cases identifiable by urine output criteria alone may have been missed. Furthermore, perioperative fluid shifts, hemodilution, and RBC transfusion may blunt early creatinine rises, potentially attenuating the sensitivity of creatinine-based AKI detection in this surgical population. Postoperative urine output data sufficient for full KDIGO-based AKI ascertainment were not available in our retrospective dataset. Fourth, EBL was estimated using a hemoglobin-based method, which may be inaccurate in the setting of major surgery with substantial fluid shifts and transfusion; accordingly, EBL should be interpreted as an approximation rather than a precise measure. Finally, intraoperative urine output was recorded as a single averaged value across the entire operative period; time-stamped data were not available, precluding assessment of duration-based oliguria or transient anuria, which may carry different prognostic implications. Despite these limitations, to our knowledge, this is the first study to examine the association between intraoperative oliguria and postoperative AKI specifically in patients undergoing hepatobiliary and pancreatic surgery managed within an ERAS protocol. The large single-center cohort with a well-defined institutional ERAS protocol allowed for consistent perioperative management across patients, reducing practice variability as a source of confounding. In addition, the inclusion of multiple ERAS-specific variables, including oral carbohydrate loading, thoracic epidural analgesia, and vasopressor use, enabled a more comprehensive assessment of the ERAS context than has been reported in prior studies.

In conclusion, this study did not find a significant association between intraoperative oliguria and postoperative AKI in patients undergoing hepatobiliary and pancreatic surgery within an ERAS protocol. These findings may prompt reconsideration of reflexive fluid administration driven solely by low urine output in this setting. However, the data do not demonstrate that fluid therapy for oliguria is ineffective or harmful, and average intraoperative urine output alone may be insufficient as the sole guide for fluid decisions. Larger prospective studies are needed before any definitive change to clinical practice can be recommended.

## Figures and Tables

**Table 1:** Baseline characteristics of patients with and without intraoperative oliguria.

Variables	Non-Oliguria (*n* = 765)	Oliguria (*n* = 51)	*p*-Value
Age, year	65 [59–71]	63 [57–72]	0.442
Sex, male	467 (61.1)	36 (70.6)	0.227
BMI, kg m^−2^	23.3 [21.2–25.5]	25.0 [22.6–26.7]	0.018 *
Diabetes	267 (34.9)	21 (41.2)	0.449
Hypertension	338 (44.2)	27 (52.9)	0.283
Ischemic cardiac disease	56 (7.3)	4 (7.8)	1.000
Cerebrovascular disease	34 (4.4)	4 (7.8)	0.440
RAS-acting agent use	166 (21.7)	15 (29.4)	0.267
Diuretic use	33 (4.3)	3 (5.9)	0.860
ASA physical status			0.800
I	31 (4.1)	2 (3.9)	
II	625 (81.7)	40 (78.4)	
III	109 (14.3)	9 (17.7)	
Preoperative laboratory data			
Hemoglobin, g dL^−1^	11.2 [10.2–12.2]	11.3 [10.2–12.4]	0.526
Creatinine, mg dL^−1^	0.77 [0.64–0.90]	0.83 [0.68–0.99]	0.019 *
Albumin, g dL^−1^	3.6 [3.3–3.8]	3.6 [3.4–3.9]	0.581

Values are expressed as median [interquartile range] or number (percentage). * *p* < 0.05. Abbreviation: BMI, body mass index; RAS: renin–angiotensin system; ASA, American Society of Anesthesiologists.

**Table 2:** Comparison between the oliguria and non-oliguria groups.

Variables	Non-Oliguria (*n* = 765)	Oliguria (*n* = 51)	*p*-Value
Surgical variables within the ERAS protocol			
Oral carbohydrate loading	657 (85.9)	40 (78.4)	0.210
Thoracic epidural analgesia	484 (63.2)	26 (51.0)	0.108
Duration of surgery, min	255 [205–307]	235 [179–312]	0.361
Type of surgery			0.223
Pancreatic resection	577 (75.4)	33 (64.7)	
Liver and bile duct surgery	163 (21.3)	16 (31.4)	
Pancreaticoduodenectomy	25 (3.3)	2 (3.9)	
Intraoperative hemodynamic data			
Lowest MBP, mmHg	60 [55–64]	61 [56–63]	0.780
Stroke volume variation, %	11.0 [8.0–13.0]	10.5 [8.0–12.0]	0.255
Intraoperative volume status			
UO, mL kg^−1^ h^−1^	0.9 [0.6–1.4]	0.2 [0.1–0.3]	<0.001
Total crystalloid, mL kg^−1^ h^−1^	7 [5–9]	6 [5–8]	0.002 *
Total colloid, mL kg^−1^ h^−1^	1 [0–2]	0 [0–1]	0.061
Total IV fluid, mL kg^−1^ h^−1^	8 [6–10]	6 [5–8]	<0.001 *
>5 mL kg^−1^ h^−1^	675 (88.2)	37 (72.6)	0.002
>8 mL kg^−1^ h^−1^	376 (49.2)	13 (25.5)	0.002
RBC transfusion	92 (12.0)	2 (3.9)	0.126
Estimated blood loss, mL	124 [0–502]	0 [0–329]	<0.001 *
Fluid balance, mL kg^−1^ h^−1^	6 [4–8]	5 [4–7]	0.056
Vasopressor infusion	500 (65.4)	26 (51.0)	0.054
AKI outcome			
AKI incidence	54 (7.1)	6 (11.8)	0.332
Stage of AKI			0.306
Stage 1	43 (5.6)	5 (9.8)	
Stage 2	7 (0.9)	0 (0.0)	
Stage 3	4 (0.5)	1 (2.0)	
Timing of AKI			0.351
Early-onset	51 (6.7)	6 (11.8)	
Late-onset	3 (0.4)	0 (0.0)	

Values are expressed as median [interquartile range], or number (percentage). * *p* < 0.05. Abbreviation: AKI, acute kidney injury; MBP, mean blood pressure; UO, urine output; IV, intravenous; RBC, red blood cell.

**Table 3:** Comparison between the AKI and non-AKI groups.

Variables	Non-AKI (*n* = 756)	AKI (*n* = 60)	*p*-Value
Baseline characteristics			
Age, year	65 [59–71]	64 [60–72]	0.818
Sex, male	454 (60.1)	49 (81.7)	0.001 *
BMI, kg m^−2^	23.3 [21.2–25.5]	24.3 [21.7–26.6]	0.058
Diabetes	265 (35.1)	23 (38.3)	0.710
Hypertension	329 (43.5)	36 (60.0)	0.019 *
Ischemic cardiac disease	54 (7.1)	6 (10.0)	0.576
Cerebrovascular disease	34 (4.5)	4 (6.7)	0.653
RAS-acting agent use	165 (21.8)	16 (26.7)	0.479
Diuretic use	31 (4.1)	5 (8.3)	0.226
ASA physical status			0.623
I	32 (4.2)	1 (1.7)	
II	615 (81.4)	50 (83.3)	
III	109 (14.4)	9 (15.0)	
Preoperative laboratory data			
Hemoglobin, g dL^−1^	11.2 [10.2–12.2]	11.7 [9.8–12.9]	0.312
Creatinine, mg dL^−1^	0.77 [0.64–0.90]	0.84 [0.68–0.92]	0.125
Albumin, g dL^−1^	3.6 [3.3–3.8]	3.6 [3.3–3.9]	0.761
Surgical variables			
Oral carbohydrate loading	657 (86.9)	49 (81.7)	0.344
Thoracic epidural analgesia	472 (62.4)	38 (63.3)	1.000
Duration of surgery, min	252 [201–304]	281 [232–358]	0.001 *
Type of surgery			0.003 *
Pancreatic resection	576 (76.2)	34 (56.7)	
Liver and bile duct surgery	156 (20.6)	23 (38.3)	
Pancreaticoduodenectomy	24 (3.2)	3 (5.0)	
Intraoperative hemodynamic data			
Lowest MBP, mmHg	60 [56–64]	57 [53–62]	0.005 *
Stroke volume variation, %	10 [8–13]	12 [9–16]	0.013 *
Intraoperative volume status			
Urine output, mL kg^−1^ h^−1^	0.8 [0.6–1.4]	0.7 [0.5–1.1]	0.069
<0.3 mL kg^−1^ h^−1^	45 (6.0)	6 (10.0)	0.332
<0.5 mL kg^−1^ h^−1^	147 (19.4)	17 (28.3)	0.137
Total crystalloid, mL kg^−1^ h^−1^	6.9 [5.4–9.2]	7.0 [4.7–9.7]	0.961
Total colloid, mL kg^−1^ h^−1^	0.5 [0.0–1.8]	1.0 [0.0–1.7]	0.154
Total IV fluid, mL kg^−1^ h^−1^	7.8 [6.1–10.1]	7.8 [5.9–11.3]	0.842
RBC transfusion	80 (10.6)	14 (23.3)	0.006 *
Estimated blood loss, mL	115 [0–497]	239 [0–508]	0.314
Fluid balance, mL kg^−1^ h^−1^	5.7 [4.1–7.9]	6.2 [4.3–10.0]	0.194
Intraoperative vasopressor use	479 (63.4)	47 (78.3)	0.028 *

Values are expressed as median [interquartile range] or number (percentage). * *p* < 0.05. Abbreviation: AKI, acute kidney injury; BMI, body mass index; RAS: renin–angiotensin system; ASA, American Society of Anesthesiologists; MBP, mean blood pressure; IV, intravenous; RBC, red blood cell.

**Table 4:** Univariable and multivariable logistic regression for predictive factors associated with acute kidney injury.

Variables	Univariable Analysis		Multivariable Analysis	
	Odds Ratio (95% CI)	*p*-Value	Odds Ratio (95% CI)	*p*-Value
Age	1.01 (0.98–1.03)	0.682		
Sex, female	0.34 (0.17–0.66)	0.002	0.28 (0.13–0.54)	<0.001
BMI	1.06 (0.99–1.14)	0.059	1.09 (1.01–1.18)	0.031
Diabetes	1.15 (0.67–1.98)	0.609		
Hypertension	1.95 (1.14–3.33)	0.015	1.64 (0.94–2.89)	0.081
ASA physical status				
I	1.00 (reference)			
II	2.60 (0.35–19.44)	0.351		
III	2.64 (0.32–21.65)	0.365		
Oral carbohydrate loading	0.67 (0.34–1.33)	0.256		
Thoracic epidural analgesia	1.04 (0.60–1.79)	0.890		
Oliguria (UO < 0.3 mL kg^−1^ h^−1^)	1.74 (0.66–4.59)	0.263	1.68 (0.60–4.01)	0.276
Preoperative serum creatinine	2.83 (0.67–11.99)	0.157		
Intraoperative vasopressor use	2.09 (1.11–3.93)	0.022	1.92 (1.03–3.83)	0.050
Total IV fluid, mL kg^−1^ h^−1^	1.03 (0.97–1.10)	0.308		
RBC transfusion	2.57 (1.35–4.88)	0.004	2.99 (1.49–5.79)	0.001

Variables with *p* < 0.1 in the univariable analysis were entered into the multivariable model. Abbreviation: CI, confidence interval; BMI, body mass index; UO, urine output; RBC, red blood cell.

## Data Availability

The data presented in this study are available on request from the corresponding author.

## Supplementary Materials

The following supporting information can be downloaded at https://www.mdpi.com/article/10.3390/jcm15135240/s1, Table S1: Components of the ERAS protocol.

## Abbreviations

The following abbreviations are used in this manuscript:

AKI	Acute kidney injury
ASA	American Society of Anesthesiologists
BMI	Body mass index
EBL	Estimated blood loss
ERAS	Enhanced Recovery After Surgery
KDIGO	Kidney Disease: Improving Global Outcomes
MBP	Mean blood pressure
RBC	Red blood cell
sCr	Serum creatinine
