# Supplementary material for: Association Between Intraoperative Oliguria and Postoperative Acute Kidney Injury in Patients Undergoing Hepatobiliary and Pancreatic Surgery Within an Enhanced Recovery After Surgery Protocol: A Retrospective Cohort Study
Source: J Clin Med. 2026 Jul 4;15(13):5240. doi: 10.3390/jcm15135240 (PMC13363048; doi:10.3390/jcm15135240)
Supplement: Supplementary file 1 [file jcm-15-05240-s001.zip › jcm-4394811-supplementary.pdf]

Intraoperative oliguria and postoperative acute kidney injury in patients undergoing hepatobiliary and pancreatic surgery within an enhanced recovery after surgery protocol: a retrospective cohort study

Running title: Intraoperative Oliguria & AKI in ERAS

Table S1. Components of the ERAS protocol.

	Components	Application
Preoperative	Preoperative counseling and education	Applied
	Restriction for perioperative biliary drainage	Applied
	Routine use of preoperative artificial nutrition	Not used
	Without bowel preparation (mechanical bowel preparation)	Applied
	Midnight carbohydrate loading	Applied
	Oral carbohydrate loading 2 h before surgery	Applied
	Antithrombotic prophylaxis	Applied
	Antibiotic prophylaxis	Applied
	Without premedication	Applied
Intraoperative	Avoiding hypothermia (Active warming with blanket-rol)	Applied
	Near-zero fluid balance	Applied
	Thoracic epidural analgesia (activated before onset of surgery and discontinued on POD 2)	Applied
	Intraoperative prophylactic prevention of postoperative nausea and vomiting	Applied
	TIVA/no inhaled anesthetics	Applied
	Maintain low FiO_2 levels to prevent hyperoxia-induced lung injury	Applied
Postoperative	Postoperative multimodal analgesia	Applied
	Prevention of postoperative nausea and vomiting using prokinetics	Applied
	Early removal of the nasogastric Tube	Applied
	Early removal of perianastomtic drains	At POD 5
	Early transurethral catheter removal	Applied
	Somatostatin analogues	Not used

	Early oral intake (postoperative artificial nutrition not routinely applied)	Applied
	Early scheduled mobilization	Applied

ERAS, Enhanced Recovery After Surgery; POD, postoperative day; TIVA, total intravenous anesthesia; FiO_2, fraction of inspired oxygen.
